# Tubular Stress Markers and Future Risk of Sepsis-Associated Acute Kidney Injury

**DOI:** 10.1016/j.xkme.2026.101314

**Published:** 2026-03-05

**Authors:** Angela K. Wong, Michael G. Shlipak, Orlando M. Gutierrez, Alexander L. Bullen

**Affiliations:** 1 The University of California, San Diego School of Medicine, San Diego, CA; 2 Kidney Health Research Collaborative, Department of Medicine, University of California, San Francisco, CA; 3 Department of Medicine, San Francisco Veterans Affairs Medical Center, San Francisco, CA; 4 Department of Medicine, University of Alabama at Birmingham, Birmingham, AL; 5 Department of Medicine, Nephrology Section, Veterans Affairs San Diego Healthcare System, La Jolla, CA; 6 Division of Nephrology and Hypertension, Department of Medicine, University of California San Diego, San Diego, CA

To the Editor:

Sepsis is a leading cause of death, especially when complicated by acute kidney injury (AKI), a common clinical syndrome linked to chronic kidney disease progression,^1^ cardiovascular disease,^2^ and mortality.^3^ Approximately two-thirds of septic shock patients develop AKI, with nearly half presenting with it upon admission.^4^^,^^5^ However, it remains unclear why some septic patients develop AKI whereas others do not.

Septic AKI is often attributed to acute tubular necrosis, in which the tubules are the primary site of kidney injury. Tubular stress markers, including tissue inhibitor of metalloproteinases 2 (TIMP2) and insulin-like growth factor binding protein 7 (IGFBP7), rise during AKI owing to impaired filtration, reduced reabsorption, and leakage from tubular damage. We investigated whether these urine stress markers, used to detect AKI acutely,^6^ can also reflect long-term tubular health and predict septic AKI when measured in the ambulatory setting.

We performed a case-control study within the Reasons for Geographical Differences in Stroke (REGARDS), a national cohort, using baseline urine samples. Patients who developed septic AKI were matched 1:1 based on age, sex, race, and time to admission with sepsis patients without AKI. We used conditional logistic regression to assess associations among TIMP2, IGFBP7, and the odds of AKI during sepsis per doubling of each biomarker, separately, and in tertiles. Detailed methods are shown in Item S1.

This study included the following 490 participants: 245 with septic AKI and 245 who were admitted with severe infection but did not have AKI. Table 1 displays participant demographics and clinical characteristics, stratified based on AKI status. The mean age was 69 ± 8 years, 44% were women, and 39% were Black. The median (interquartile range) baseline estimated glomerular filtration rate (eGFR) for cases and controls was 73 (55-90) and 82 (65-92) mL/min/1.73 m^2^, respectively, whereas the median (interquartile range) albuminuria was 19 (8-87) versus 9 (5-22) mg/g. The median (interquartile range) time between the REGARDS baseline visit and their index hospitalization was 4.3 (2.5-6) years. Compared with controls, cases more often had diabetes, a lower eGFR, and higher albuminuria at baseline. During hospitalization for sepsis, cases had higher Sequential Organ Failure Assessment scores and mortality. TIMP2 was not, whereas IGFBP7 was, associated with a higher risk of septic AKI in the fully adjusted analysis (odds ratio, 1.27; 95% confidence interval, 1.01-1.60), which included the use of angiotensin-converting enzyme inhibitor, angiotensin II receptor blocker, high-sensitivity C-reactive protein, Sequential Organ Failure Assessment score, hospital mortality, baseline eGFR, and urine albumin, as shown in Table 2. In sensitivity analyses, we indexed each biomarker to urine creatinine, rather than adjusting for urine creatinine as a separate covariate, yielding similar results (Table S1).

**Table 1:** Baseline Characteristics of REGARDS Participants With Sepsis Based on AKI Status

	Controls (n = 245)	Cases (n = 245)
Age, y (SD)	69 (8)	69 (8)
Female, n (%)	107 (44)	107 (44)
Race, n (%)		
Black	96 (39)	96 (39)
White	149 (61)	149 (61)
Diabetes mellitus, n (%)	81 (33)	108 (44)
Mean systolic BP, mm Hg (SD)	131 (19)	133 (18)
Mean diastolic BP, mm Hg (SD)	75 (10)	75 (11)
Use of ACEi, n (%)	78 (32)	91 (37)
Use of ARBs, n (%)	41 (17)	59 (24)
Use of mineralocorticoids, n (%)	10 (4)	11 (4)
Use of NSAID, n (%)	51 (21)	62 (25)
Admission destination, n (%)		
ICU	23 (10)	44 (18)
General ward	196 (84)	185 (78)
Other/unknown	26 (11)	16 (7)
Type of infection		
Respiratory	110 (45)	120 (49)
Urinary tract	34 (14)	36 (15)
Abdominal	53 (22)	37 (15)
Skin	22 (9)	23 (9)
Other	26 (11)	28 (12)
Mean hsCRP at baseline, (SD)	0.51 (0.50)	0.56 (0.50)
SOFA score		
0	45	23
1	69	31
2	54	55
3-4	52	64
≥5	25	72
Hospital mortality, n (%)	10 (4.1)	56 (22.9)
Median eGFR, (IQR)	82 (65-92)	73 (55-90)
Median urine ACR, mg/g (IQR)	9 (5-22)	19 (8-87)
Median urine TIMP2, ng/mL (IQR)	1.4 (0.7-2.6)	1.5 (0.8-2.7)
Median urine IGFBP7, ng/mL (IQR)	99.6 (56.5-166.1)	106.4 (55.1-197.6)

Abbreviations: ACEi, angiotensin-converting enzyme inhibitors; ACR, albumin-creatinine ratio; AKI, acute kidney injury; ARBs, angiotensin II receptor blockers; BP, blood pressure; eGFR, estimated glomerular filtration rate; hsCRP, high-sensitivity C-reactive protein; ICU, intensive care unit; IGFBP7, insulin-like growth factor binding protein 7; IQR, interquartile range; NSAID, nonsteroidal anti-inflammatory drugs; REGARDS, Reasons for Geographical Differences in Stroke; SD, standard deviation: SOFA, Sequential Organ Failure Assessment; TIMP2, tissue inhibitor of metalloproteinases 2.

**Table 2:** Association of Urine Tubular Stress Markers With Risk of Septic Acute Kidney Injury Adjusted to Urine Creatinine

	No. of Cases	TIMP2		No. of Cases	IGFBP7	
		Model 1^a^	Model 2^b^		Model 1^a^	Model 2^b^
		OR (95% CI)	OR (95% CI)		OR (95% CI)	OR (95% CI)
Per doubling	245	1.13 (0.96-1.34)	1.12 (0.91-1.39)	245	1.22 (1.02-1.46)	1.27 (1.01-1.60)
Tertile 1	81/158	Reference	Reference	76/153	Reference	Reference
Tertile 2	61/138	0.84 (0.51-1.37)	0.82 (0.43-1.57)	58/135	0.87 (0.51-1.48)	0.85 (0.44-1.66)
Tertile 3	103/194	1.43 (0.80-2.57)	1.13 (0.53-2.43)	111/202	1.52 (0.89-2.60)	1.80 (0.90-3.61)

*Note*: Tertiles were created from the controls.

Abbreviations: CI, confidence interval; DM, diabetes mellitus; eGFR, estimated glomerular filtration rate; IGFBP7, insulin-like growth factor binding protein 7; OR, odds ratio; SOFA, Sequential Organ Failure Assessment; TIMP2, tissue inhibitor of metalloproteinases 2.

a Model 1: urine creatinine, DM, systolic blood pressure, and diastolic blood pressure.

b Model 2: model 1 + angiotensin-converting enzyme inhibitors, angiotensin II receptor blockers, C-reactive protein, SOFA score, hospital mortality, baseline eGFR, and urine albumin.

Our findings align with the evidence from other tubular biomarkers supporting the role of baseline tubular health in predicting kidney vulnerability to future insults. Urine uromodulin (uUMOD), the most abundant urine protein in healthy adults, is synthesized exclusively in the thick ascending limb of Henle loop and the distal convoluted tubule of the kidney.^7^ Higher uUMOD levels may serve as a surrogate for kidney tubule functional capacity.^7^ We have previously demonstrated that lower uUMOD and higher tubular reabsorption marker concentrations, such as alpha-1 microglobulin, are associated with increased risk of AKI, independent of eGFR and albuminuria.^8^ However, this study involved participants in the Systolic Blood Pressure Intervention Trial, in which over 80% of AKI events were linked to intravascular volume depletion or hypotension.^9^ In a subsequent study, we found no association between uUMOD or reabsorption markers and septic AKI.^10^ The pathophysiology of septic AKI is complex and includes intrarenal hemodynamic changes, endothelial dysfunction, infiltration of inflammatory cells in the renal parenchyma, intraglomerular thrombosis, and obstruction of tubules with necrotic cells and debris. In this study, IGFBP7 was associated with higher odds of septic AKI, suggesting that tubular dysfunction may increase susceptibility to septic AKI, despite being measured over 4 years before the insult. Prior research has highlighted the predictive value of tubular stress markers in acute settings such as surgery and critical illness. To the best of our knowledge, this study is the first to demonstrate their prognostic use when measured years before hospitalization.

This study demonstrates that abnormalities in tubular stress markers can be detected long before AKI occurs, suggesting a role for routine testing to complement eGFR and albuminuria in risk stratification. Whereas IGFBP7 emerged as a predictor, TIMP2 did not, potentially owing to differences in proximal versus distal tubular origin. However, when assessing the functional form of association based on tertiles, higher levels of both stress markers were associated with higher odds of AKI.

Strengths of this study include a large cohort with a significant prevalence of diabetes and hypertension, key risk factors for renal complications, and detailed information during hospitalization. Limitations include reliance on single baseline samples and the lack of long-term follow-up to assess prognosis in participants with higher levels of tubular stress markers.

In summary, when measured in high-risk community-dwelling patients during periods of health, a tubular stress marker, IGFBP7, was significantly associated with the future risk of septic AKI. These findings support our original hypothesis that markers of tubule dysfunction measured during times of health are associated with subsequent sepsis-associated AKI. More extensive studies could be considered to evaluate the use of IGFBP7 in screening individuals at high risk for AKI, in which preventive strategies may lead to a lower incidence of AKI.

## Supplementary Materials

Supplementary File (PDF) Item S1; Table S1.
