# Supplementary material for: Tubular Stress Markers and Future Risk of Sepsis-Associated Acute Kidney Injury
Source: Kidney Med. 2026 Mar 5;8(5):101314. doi: 10.1016/j.xkme.2026.101314 (PMC13125156; doi:10.1016/j.xkme.2026.101314)
Supplement: Supplementary File (PDF) — Item S1; Table S1. [file mmc1.pdf]

Item S1. Detailed Methods

Study Design and Participants

REGARDS is a population-based longitudinal cohort study of adults recruited from across the United States. Details of the study have been published previously.[1]In brief, the study consists of adults aged 45 years and older and was designed to investigate the reasons for higher stroke mortality among Black compared to White adults, as well as among adults residing in the Southeast region of the United States.[1,2]Between January 2003 and October 2007, 30,239 participants were recruited. The institutional review boards of the participating institutions approved the REGARDS study, and all participants provided verbal consent before the telephone interview and written informed consent before completing the in-home study visit. Trained staff collected baseline information on REGARDS participants during a preliminary phone interview and an in-home physical examination. The in-home visit included blood pressure measurements, anthropometric measurements, and the collection of blood and spot urine specimens. Participants were instructed to fast for 10 to 12 hours before the in-home visit. Participants provided written informed consent.

REGARDS followed all participants prospectively and captured information about emergency room visits and hospitalizations, among which hospital records were retrieved and stored. For this ancillary study, we first identified all the participants who were hospitalized with a severe infection in addition to systemic inflammatory response syndrome criteria, including (1) heart rate >90 beats/min, [3] fever (temperature >38.3 or <36°C), (3) tachypnea (>20 breaths/min) or PCO2 <32 mm Hg, and (4) leukocytosis (white blood cells >12,000 or <4,000 cells/mm3 or >10% band forms) during follow-up after their baseline REGARDS visit.[4] Two trained abstractors independently reviewed all relevant medical records to identify clinical and

laboratory information, confirm the presence of a severe infection on initial hospital presentation, and verify the relevance of the severe infection as a significant reason for hospitalization. They also abstracted daily serum creatinine values from the hospital records, which were used to categorize AKI events. Adjudication captured hospitalizations from February 5, 2003, through December 31, 2012. An initial review of 1,349 hospital records indicated excellent interrater agreement for the presence of a severe infection ($\kappa = 0.92$).[5] Cases were matched (1:1) by age (+/- 5 years), sex, race, and the number of days from the baseline REGARDS visit to hospitalization due to sepsis (+/- 90 days) with individuals admitted with sepsis but without AKI (controls).

According to the REGARDS policy, the aims and analysis plan for this manuscript were prespecified and reviewed and approved by the REGARDS publications committee, which also reviewed the final manuscript and assured the study protocol was followed. The institutional review board at UC San Diego and the Veterans Affairs San Diego Healthcare System approved the study.

Covariates

At the baseline REGARDS visit, age, sex, and race were obtained by self-report. REGARDS obtained an inventory of all medications used by participants at baseline. Diabetes mellitus was defined as a fasting glucose ≥ 126 mg/dL, non-fasting glucose ≥ 200 mg/dL, or self-reported use of anti-diabetic medications. Hypertension was defined as either self-reported use of antihypertensive drugs or measured systolic blood pressure ≥ 140 mm Hg, or diastolic blood pressure ≥ 90 mm Hg measured during the home examination. High-sensitivity C-reactive protein (hsCRP) was determined using particle-enhanced immunonephelometry (N hsCRP,

Siemens AG, Munich, Germany). We defined hsCRP >3.0 mg/dl as abnormal, consistent with prior studies.[6]

Additional variables obtained during hospitalization included infection type and the Sequential Organ Failure Assessment (SOFA) score for the cardiovascular, respiratory, renal, hepatic, hematologic, and neurologic systems.

Urine Biomarker Measurements

Spot urine samples were collected during the REGARDS baseline visit, centrifuged and aliquoted, then sent to the central laboratory at the University of Vermont on ice overnight. They were stored at -80° Celsius until thawed for biomarker measurements. The biomarkers were measured without prior thawing at the UAB-UCSD O'Brien Center for Acute Kidney Injury Research. Ten percent of the samples were measured in duplicate to assess precision. Laboratory personnel conducting the biomarker assays were blinded to clinical information.

Biomarker Measurements

Urine TIMP-2 and IGFBP7 were measured using sandwich immunoassays (ELISA; R&D Systems, Minneapolis, MN, for TIMP-2 and Boster Biological Technology, Pleasanton, CA, for IGFBP7, respectively) following the manufacturer's instructions. The analytic ranges for TIMP2 and IGFBP7 were 0.02-14.1 ng/mL and 3.3-15.5 ng/mL, respectively. The coefficient of variation (CV) for TIMP2 was 2.5-3.4% and 12-13.5% for IGFBP7. Urine creatinine concentration was measured using the rate Jaffe method (Roche/Hitachi, Basel, Switzerland).

Acute Kidney Injury

AKI was defined using the Kidney Disease Improving Global Outcomes (KDIGO) criteria[7] and required a rise in serum creatinine from hospital admission of 0.3 mg/dL or an increase in serum creatinine of 1.5 times the admission value. Urine output data were not reliably

available in hospital records and were therefore not utilized for AKI criteria. We used the first serum creatinine (sCr) measurement during hospitalization as the baseline value since we did not have access to outpatient sCr measurements except at the time of the baseline REGARDS visit, which occurred at variable time points, with a median elapsed time of 4.3 years between study entry and index admission. This approach is consistent with the most recent consensus definition that proposes a rolling 48-hour window for AKI ascertainment during hospitalization.[7] To assess the increase in creatinine, we used all sCr values during the first seven days of hospitalization. We excluded participants with fewer than two sCr measurements during hospitalization, as well as those with a history of requiring dialysis or receiving a kidney transplant.

Overall, 1557 REGARDS participants were admitted with sepsis during follow-up. Among these, 245 individuals (15.7%) had AKI events during their hospitalizations.[5]

Statistical Analysis

We first divided participants into those who experienced septic AKI and those who did not. We compared baseline characteristics between these groups using the chi-squared test for categorical variables and the Wilcoxon rank-sum test for continuous variables.

Given the skewed distributions of the biomarkers, we log-base-2 transformed each biomarker to facilitate the interpretation of parameter estimates as "per two-fold higher" levels of each biomarker. Using conditional multivariate logistic regression, we evaluated the association of TIMP2 and IGFBP7 at baseline with the odds of AKI. An initial model was adjusted for urine creatinine to address urine tonicity at the time of biomarker urine collection, along with systolic and diastolic blood pressures and diabetes mellitus. Model 2 additionally adjusted for angiotensin-converting enzyme inhibitors, angiotensin II receptor blockers, high-sensitivity C-reactive protein, SOFA score, hospital mortality, baseline eGFR, and urine albumin. We also

conducted our analysis, indexing the biomarkers to urine creatinine rather than adjusting for it. [8] The main models of interest evaluated each biomarker on a continuous scale (per 2-fold increase). To assess the functional form of the associations, we also evaluated them by tertiles, using the lowest tertile as the reference category. To avoid biasing tertile cut-off points for those who would later develop septic AKI, we chose to define tertiles among the controls. These same cut-off points were then used to assign cases to the tertile groups. When associations changed monotonically across tertiles, we focused our interpretation on the results of the continuous models to maximize power.

All analyses were conducted using Stata/MP Version 15.1 (StataCorp LCC, College Station, TX). P values <0.05 were considered statistically significant for all analyses.

Supplementary References

1. Howard VJ, Cushman M, Pulley L, et al. The reasons for geographic and racial differences in stroke study: objectives and design. *Neuroepidemiology*. 2005;25(3):135-43. doi:10.1159/000086678

2. Warnock DG, McClellan W, McClure LA, et al. Prevalence of chronic kidney disease and anemia among participants in the Reasons for Geographic and Racial Differences in Stroke (REGARDS) Cohort Study: baseline results. *Kidney international*. Oct 2005;68(4):1427-31. doi:10.1111/j.1523-1755.2005.00553.x

3. Bignall ONR, 2nd, Crews DC. Stony the road we trod: towards racial justice in kidney care. *Nat Rev Nephrol*. Feb 2021;17(2):79-80. doi:10.1038/s41581-020-00389-w

4. Levy MM, Fink MP, Marshall JC, et al. 2001 SCCM/ESICM/ACCP/ATS/SIS International Sepsis Definitions Conference. *Crit Care Med*. Apr 2003;31(4):1250-6. doi:10.1097/01.Ccm.0000050454.01978.3b

5. Wang HE, Powell TC, Gutiérrez OM, Griffin R, Safford MM. Prehospitalization Risk Factors for Acute Kidney Injury during Hospitalization for Serious Infections in the REGARDS Cohort. *Nephron extra*. Sep-Dec 2015;5(3):87-99. doi:10.1159/000441505

6. Wang HE, Shapiro NI, Safford MM, et al. High-sensitivity C-reactive protein and risk of sepsis. *PLoS One*. 2013;8(7):e69232. doi:10.1371/journal.pone.0069232

7. KDIGO. Clinical Practice Guideline for Acute Kidney Injury. Section 2: AKI Definition. *Kidney international Supplement*. 2012;2:19-36.

8. Koopman JJE, Scherzer R, Ix JH, Shlipak MG, Waikar SS. A Comparison of Different Estimates of Albuminuria in Association with Mortality in Epidemiologic Research. *Clinical*

journal of the American Society of Nephrology : CJASN. Dec 7 2020;15(12):1814-1816. doi:10.2215/cjn.07290520

Table S1. Association of urine tubular stress markers indexed to urine creatinine with risk of septic acute kidney injury

	**Indexed to urine creatinine**	
	**model 1†**	**model 2††**
	**OR (95% CI)**	**OR (95% CI)**
Urine TIMP2	1.18 (0.97, 1.42)	1.19 (0.93, 1.53)
Urine IGFBP7	1.26 (1.00, 1.60)	1.38 (1.08, 1.76)

Abbreviations: TIMP2, tissue inhibitor of metalloproteinases-2; IGFBP7, insulin-like growth factor binding protein-7; DM, diabetes mellitus; eGFR, estimated glomerular filtration rate

† Model 1: DM, systolic blood pressure, diastolic blood pressure

†† Model 2: model 1 + angiotensin-converting enzyme inhibitors, angiotensin II receptor blockers, C-reactive protein, SOFA score, hospital mortality, baseline eGFR and urine albumin
